# Delivering Care Consistent With the Psychosocial Standards—Provider Report: Implementing the Standards Together—Engaging Parents and Providers in Psychosocial Care (iSTEPPP) Study

**DOI:** 10.1002/pbc.31959

**Published:** 2025-08-19

**Authors:** Anne E. Kazak, Michele A. Scialla, Kimberly S. Canter, Victoria Sardi-Brown, Kimberly Buff, Kamyar Arasteh, Emily Pariseau, Eric Sandler, Lori Wiener

**Affiliations:** 1 Nemours Children’s Health, Wilmington, Delaware, USA; 2 Sidney Kimmel Medical College of Thomas Jefferson University, Philadelphia, Pennsylvania, USA; 3 Mattie Miracle Cancer Foundation, Arlington, Virginia, USA; 4 Momcology, St. Paul, Minnesota, USA; 5 National Cancer Institute, Bethesda, Maryland, USA

**Keywords:** pediatric oncology, psychosocial, standards of care

## Abstract

**Background:**

Evidence-based Standards for Psychosocial Care for Children with Cancer and their Families were published in 2015. Determining how often care delivery practices and approaches align with the Standards is important for understanding the reach of the Standards.

**Procedure:**

Medical (*n* = 73) and psychosocial (*n* = 99) providers from 129 of 197 (65%) Children’s Oncology Group (COG) programs in the United States completed an online survey. Participants reported the frequency, timing, and psychosocial care delivery approaches for each Standard. Program size was considered, and the data were compared with those from a similar 2016 survey.

**Results:**

Using a 5-point scale, services consistent with the Standards are provided across programs—mean frequencies of 3.29–4.61 (5-point scale). Standards related to screening and monitoring (*M* = 4.42) are delivered more often than Standards related to intervention and support (*M* = 4.25), supportive care and bereavement (*M* = 3.88), and family and community (*M* = 3.72) (*p* < 0.001). Ratings of quality of care are near the midpoint of the scale. Findings are consistent with a 2016 report, although care related to family and community support was less frequent in 2023. Program size and provider discipline were not associated with quality or frequency.

**Conclusions:**

Providers report that psychosocial care aligned with the Standards is generally provided and perceive quality of care positively. Variability in types of care, frequency, and quality, and lack of change from when the Standards were first published, highlight the importance of improving the implementation of evidence-based approaches.

## 1 Introduction

Psychosocial support for children with cancer and their families is an essential component of comprehensive care [1–3]. The Standards for Psychosocial Care for Children with Cancer and Their Families (Standards [4]), were developed by more than 80 multidisciplinary pediatric oncology professionals and patient and family advocates with support from the Mattie Miracle Foundation (www.mattiemiracle.com). Each Standard is based on a rigorous, systematic literature review, including independent appraisal of the body of evidence using the Grading of Recommendations Assessment, Development, and Evaluation (GRADE) system.

**FIGURE 1:**
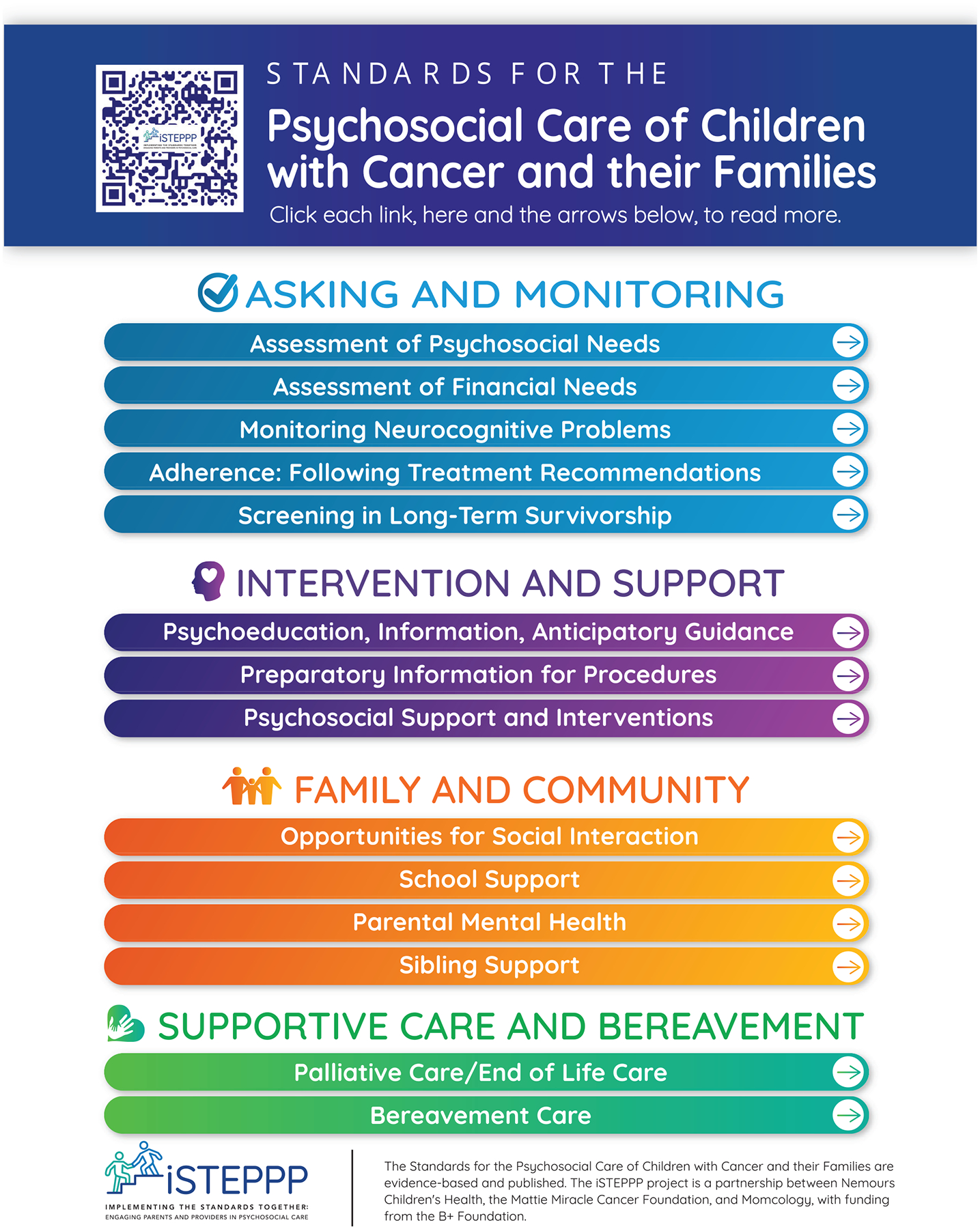
Standards for the psychosocial care of children with cancer and their families.

Fourteen (the 15th standard is not discussed in this paper as it pertains to training, credentialing, supervision, and other aspects of professional practice) Standards are organized in four groups (Figure 1): (i) Asking and Monitoring: Assessment of psychosocial needs (PSS1 [5]); assessment of financial needs (PSS5 [6]); monitoring of neurocognitive problems (PSS2 [7]); facilitating adherence to treatment (PSS12 [8]); and screening in long-term survivorship (PSS3 [9]). (ii) Intervention and Support: Psychoeducation, information and anticipatory guidance (PSS7 [10]); preparatory information for procedures (PSS8 [11]); and psychosocial support and interventions (PSS4 [12]). (iii) Family and Community: opportunities for social interaction (PSS9 [13]); school support (PSS11 [14]); parental mental health (PSS6 [15]); and sibling support (PSS10 [16]). (iv) Supportive Care and Bereavement: Palliative care/end-of-life care (PSS13 [17]); and bereavement care (PSS14 [18]). The full set is in Supporting Information S1.

After the publication of the Standards, a survey of Children’s Oncology Group (COG) sites across the United States (US), the Preparing to Implement the Standards: Psychosocial Services and Staffing (PIPS-CSS) study [19], showed most programs had a social worker and a child life specialist, although the full complement of psychosocial staff members was not consistently available. Overall, psychosocial care was provided for each Standard; however, the approaches and the extent to which care was delivered to all patients and families varied [20]. An institutional scoring system (Matrix) and accompanying Guidelines [21] were published and used to evaluate the inclusion of each Standard and improve implementation [22, 23].

The current study, *Implementing the Standards Together—Engaging Parents and Providers in Psychosocial care* (iSTEPPP), aims to further accelerate implementation of approaches aligned with the Standards by assessing practice change since the Standards were published. The staffing data from iSTEPPP are similar to 2016 PIP-CSS data, but show some incremental increases in the size of the psychosocial team (e.g., social work, psychology, neuropsychology, psychiatry), and an increase in Spanish-speaking psychosocial staff [24].

In the current paper, we use iSTEPPP data to describe how medical and psychosocial providers in pediatric oncology treatment programs in the United States report providing psychosocial care aligned with the Standards, and detail the timing and approaches utilized. We examined differences by the size of program, type of provider, and compared PIPS-CSS and iSTEPPP data to assess changes from 2016 to 2023.

## 2 Method

### 2.1 Study Design, Sample, and Recruitment

The iSTEPPP study is a collaborative research initiative with pediatric oncology patient and family advocates [25]. The sample description, recruitment, survey questionnaire development, and data on the size of the programs are described in Scialla et al. [24]. All study procedures were reviewed by the Nemours Institutional Review Board (IRB), which granted a waiver of documentation of informed consent and determined the study to be exempt from further IRB review. The data in the current paper are based on survey questions that are identical to the PIPS-CSS study [20]. Using the same survey questions allowed for comparisons across time, an a priori goal of the study.

### 2.2 Measures

#### 2.2.1 Overall Quality of Care—Provider Report

Psychosocial and medical providers responded to an item about the overall quality of psychosocial care (“The psychosocial care that pediatric cancer patients/families receive in our program is comprehensive” and “state of the art.”) on a 5-point scale from “strongly disagree” (1) to “strongly agree” (5), with a midpoint (3) indicating “neutral.”

#### 2.2.2 Implementation of the Standards—Provider Report

Psychosocial and medical providers (one of each per site) responded to a statement about psychosocial care for each Standard, indicating how often these services are provided on a 5-point scale, from Never (1) to Always (5), with the midpoint (3) indicating “sometimes.”

#### 2.2.3 Approaches Used and Timing of Care—Provider Report

Psychosocial providers indicated specific approaches used to meet the Standards. They could check all responses that applied (e.g., structured interview, questionnaire). Participants could also write in how they met each Standard. For most of the Standards, they also selected when the services were provided (e.g., at diagnosis, within the first week).

### 2.3 Data Analysis

Survey responses were collected in REDCap [26]) and imported into SPSS [27]. All analyses were conducted using SPSS and Stata 18.5 (StataCorp LLC, College Station, Texas). We first reported the broadest evaluation of psychosocial care, whether providers perceive care as comprehensive and state-of-the-art. We used a Likert-type scale from 1 (strongly disagree) to 5 (strongly agree) for the full sample and medical and psychosocial providers separately. A Mann–Whitney *U* test assessed the differences between medical and psychosocial providers.

The frequency of care delivery of each of the 14 Standards was rated on a 1 (never) to 5 (always) Likert-type scale. Mean frequencies for each Standard were calculated for the full sample, and medical providers and psychosocial staff separately. Differences in mean frequencies for each Standard based on the discipline of the respondent were tested using the Mann–Whitney *U* test, correcting for multiple comparisons by Bonferroni adjustment of *p* < 0.01. Mean frequencies for the four groups of Standards (Asking and Monitoring, Intervention and Support, Family and Community, Supportive Care and Bereavement) were compared using the Skillings–Mack test and adjusting *p*-values with the Bonferroni method for multiple comparisons.

We examined the association of the size of the program (number of new patients in 2022) with provider perceptions of care as comprehensive and with the rating of each Standard using multilevel ordered logistic regression models. We adjusted the criterion for multiple comparisons to a *p*-value of 0.01. To understand differences between the 2016 PIPS-CSS data and the 2023 iSTEPPP data, responses to questions related to the comprehensiveness of care and the frequency of care provided for each Standard were tested using the Mann–Whitney *U* test.

## 3 Results

### 3.1 Participants

**FIGURE 2:**
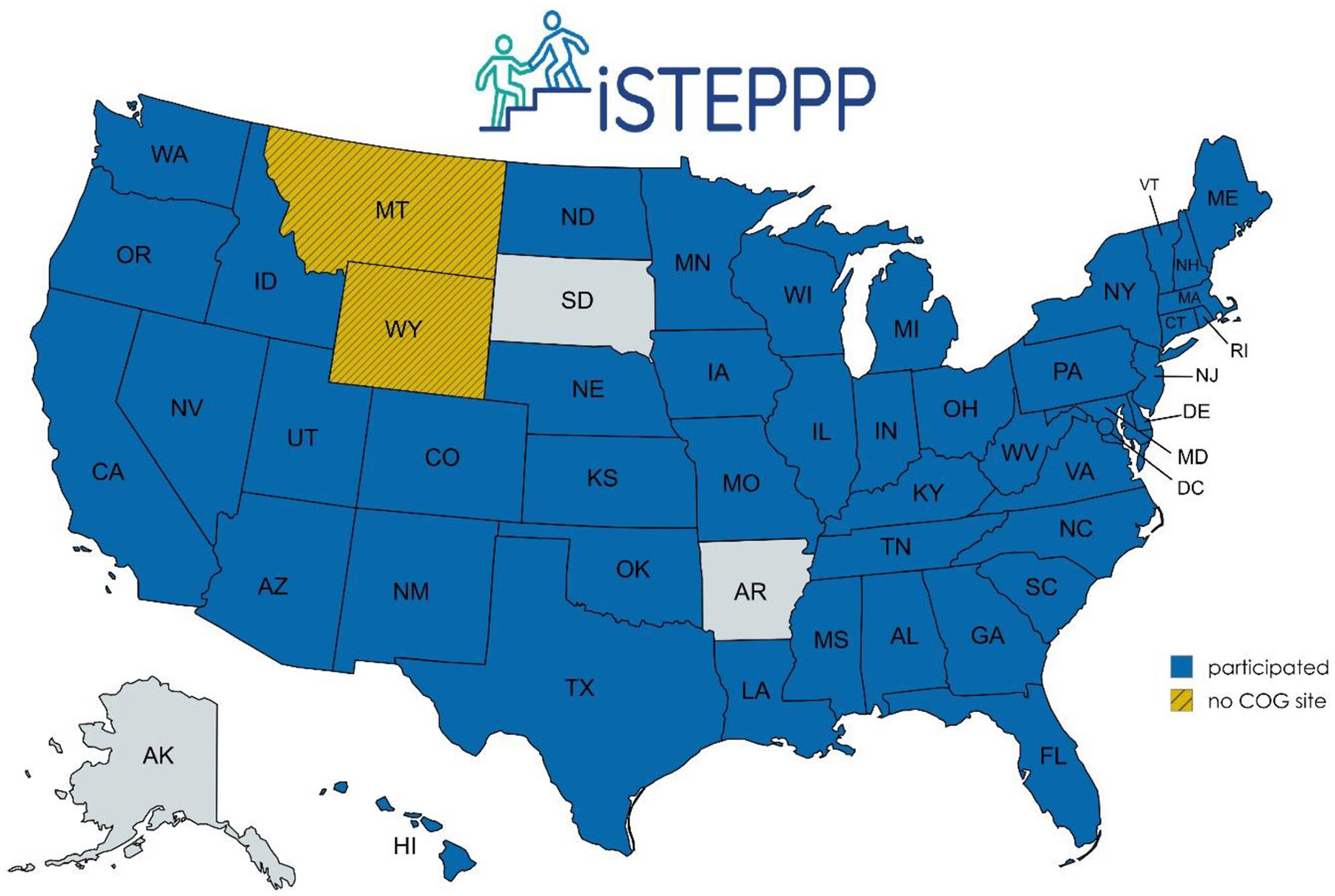
Map of the United States showing COG (Children’s Oncology Group) site and iSTEPPP (Implementing the Standards Together—Engaging Parents and Providers in Psychosocial Care) participation status.

Participants were medical (*n* = 69) and psychosocial (*n* = 99) providers representing 129 of 197 US COG pediatric oncology treatment programs ([28]; institutional response rate 65.5%) from 45 geographically dispersed states and the District of Columbia (Figure 2). Each program identified up to two participants (one medical and one psychosocial provider) to complete the survey. Fifty-four programs (41.9%) returned data from both a medical and a psychosocial provider. Programs of all sizes (number of new patients in 2022): <50 (*n* = 33, 27%); 50–100 (*n* = 45, 36%); 101–250 (*n* = 30, 25%); >250 (*n* = 12, 10%) were represented.

### 3.2 Quality of Psychosocial Care

**FIGURE 3:**
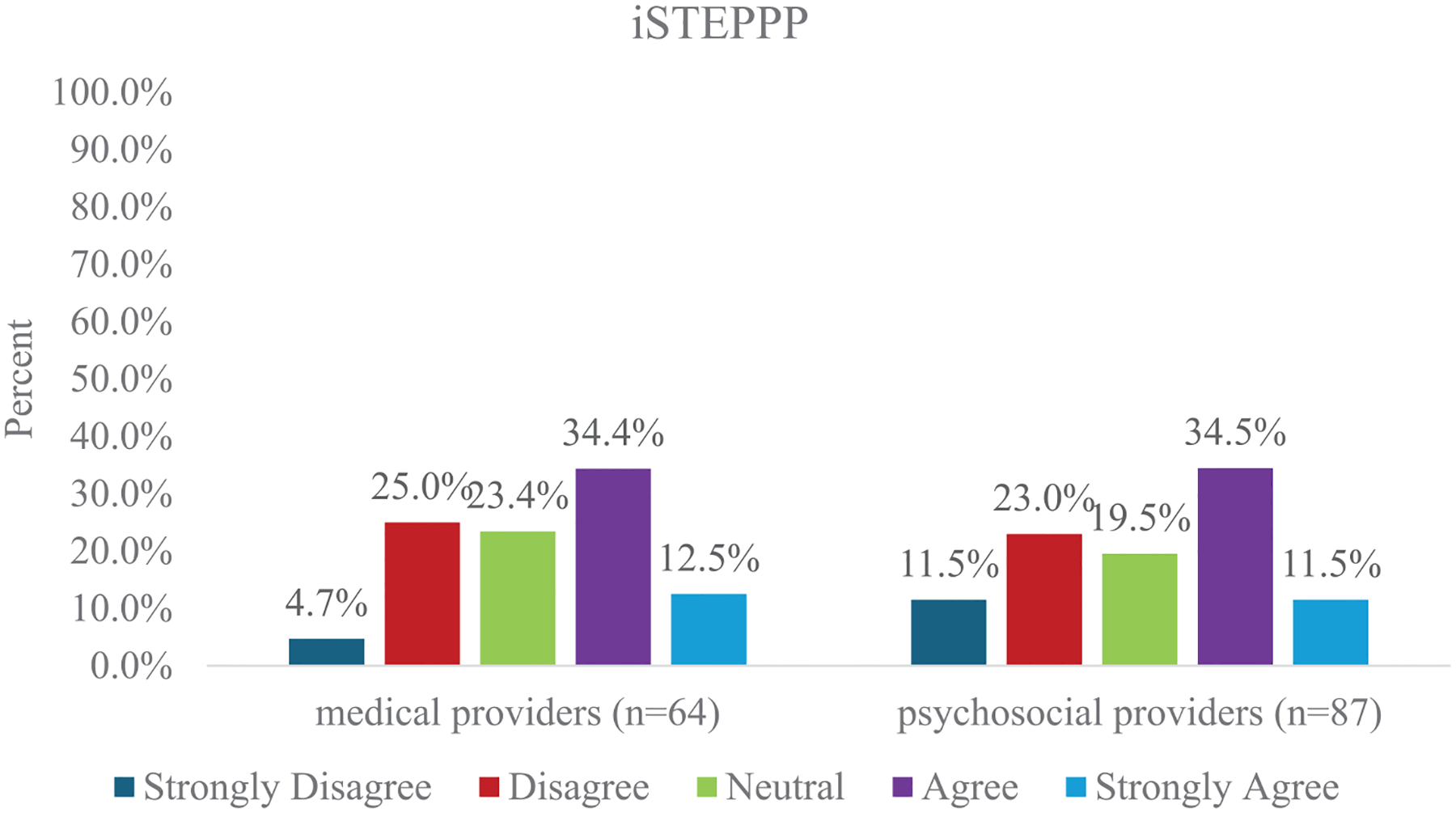
Endorsement of “comprehensive and state-of-the-art” care by medical and psychosocial providers.

With respect to whether psychosocial care in their programs was comprehensive and state-of-the-art, ratings of medical and psychosocial providers were not significantly different. The means for both groups were near the midpoint of 3 on the 5-point scale of 1 (strongly disagree) to 5 (strongly agree): 3.25 (2.98–3.53) for medical, 3.11 (2.86–3.37) for psychosocial providers, and 3.17 (2.98–3.36) for the full sample. There was, however, notable variability in the responses to this item (Figure 3). While close to half of the medical (46.9%) and psychosocial (46.0%) providers indicated that they “agree” or “strongly agree” with this statement, about one-third of both medical (29.7%) and psychosocial (34.5%) providers indicated that they “disagree” or “strongly disagree” (Figure 2). There was no significant association of program size and provider ratings of quality of care (*p* > 0.01).

### 3.3 Mean Frequencies of Care Provided by Each Standard

The mean frequencies across the Standards ranged from 3.29 (sometimes) to 4.61 (usually) on a 1 (never) to 5 (always) scale (Table 1). There were no differences between medical and psychosocial providers (*p*-values >0.01). There was, however, variability in mean frequency for the four groups of Standards (Skillings–Mack = 196.58, *p* < 0.001). Standards related to Asking and Monitoring (*M* = 4.42) are delivered more often than Intervention and Support (*M* = 4.25), Supportive Care and Bereavement (*M* = 3.88), and Family and Community (*M* = 3.72).

There were no significant associations of program size and frequency of care provided (*p*-values > 0.01). Table 2 summarizes approaches and timing of care by Standard.

### 3.4 Asking and Monitoring

For the five Standards related to Asking and Monitoring, informal discussion or verbal reports were the most frequently endorsed approach (73.4%–85.3%). Validated screening tools were utilized less frequently. For example, 25.7% endorsed using the Psychosocial Assessment Tool [29] and 18.3% the Distress Thermometer [30]. The most frequent time for providing care was when a problem was identified for PSS1 (79.8%) and PSS2 (74.3%), and the first week after diagnosis for PSS5 (62.4%).

### 3.5 Intervention and Support

Informal discussions and supportive psychotherapy were frequently mentioned approaches. With respect to specific evidence-based approaches, there was endorsement of cognitive behavioral therapy across these Standards (66.1%–69.7%), family therapy (33.9%), and cancer-specific approaches such as Problem-Solving Skills Training [31] (35.8%), Surviving Cancer Competently Intervention Program (SCCIP; SCCIP-ND; [32, 33]) (4.6%). Psychopharmacologic treatment (i.e., psychotropic medication) was reported by 61.5%, reflecting its use for pre-existing mental health concerns or adverse psychological effects related to oncologic treatment for children and adults [34, 35]. Care is provided most often when problems are identified (79.8%), followed by within a week of diagnosis (66.1%). Care is less often provided at every inpatient encounter (41.3%) and at every outpatient visit (25.7%). With respect to psychoeducation and procedural support, sessions with individual patients are common across these Standards (67.0%–81.7%), along with joint sessions with family (81.7%–86.2%). Distraction (84.4%) and relaxation (79.8%) are often used for procedures. Written materials (61.5%–80.7%) augment interventions.

### 3.6 Family and Community

Programs reported providing patients with opportunities to connect with others via community-based camps (68.8%), support groups (22.9%), online chats (20.2%), and other programs (22.9%). Most cancer programs have a school liaison (68.8%) and/or a hospital-based school program (45.9%). Caring for the mental health needs of parents was accomplished primarily by informal discussion (80.7%), general psychological treatments, and referral to community providers (60.6%–76.1%). Similar to other Standards, identified concerns prompt intervention (71.6%). Sibling issues are most often handled in informal discussions (78.0%) and by referral to community providers (66.1%).

### 3.7 Supportive Care and Bereavement

The services related to the supportive care and bereavement Standards were noted to involve other hospital services or referral to community agencies, as the primary providers. Most programs send a card or letter (67.9%), or (57.8%) make a call after a child dies. Most have a hospital memorial service (54.1%). In-person meetings (20.2%), psychotherapy (18.3%), or support groups (21.1%) were less commonly offered.

### 3.8 Differences in Frequency of Care Provided: PIPS-CSS Versus iSTEPPP

The frequency of care provided between 2016 and 2023 (PIPS-CSS, iSTEPPP) was not significantly different for the individual Standards related to Asking and Monitoring, Intervention and Support, and Supportive Care and Bereavement (*p*-values >0.01) (Table 3). Three of the four Standards related to Family and Community showed a significant decrease in the frequency with which care was provided in iSTEPPP as compared to previously: Parental Mental Health PSS6 (*M* = 4.23, 95% CI = 4.13–4.33; *M* = 3.84, 95% CI = 3.69–3.99, respectively), Opportunities for Social Interaction PSS9 (*M* = 3.96, 95% CI = 3.85–4.07; *M* = 3.46, 95% CI = 3.34–3.58, respectively), and Siblings PSS10 (*M* = 3.65, 95% CI = 3.53–3.76; *M* = 3.29, 95% CI = 3.15–3.42, respectively).

## 4 Discussion

There is robust evidence for psychosocial care that encompasses a broad array of patient and family concerns, and evidence-based Standards that guide the provision of this care [4]. We conducted a national survey (in 2023) to determine staffing and services in collaboration with patient and family advocates [25]. We also had the unique opportunity to compare our current findings with those from a previous study [19, 20].

Medical and psychosocial providers agree that comprehensive and state-of-the-art care aligned with the Standards is provided at their sites, with the average level of endorsement being around the midpoint of 5-point scales. Thus, on average, providers appraised the quality of care as “neutral” and indicated it was “sometimes” provided. There is considerable variability, and approximately one-third of programs reported that they were not providing comprehensive care across the realms covered by the full set of Standards. Closely observing the individual Standards and the frequency and timing of the care provided gives valuable information. Care provided relevant to the four Standards associated with Asking and Monitoring (psychosocial assessment, financial need, survivorship, adherence, and neurocognitive testing) was more often provided than those Standards related to Intervention and Support, Family and Community, and Supportive Care and Bereavement.

With respect to Asking and Monitoring, assessing psychosocial well-being and risk is a critical first step to help show areas of need for the patient and family, thereby offering insights into specific interventions that can be provided for patients. There are evidence-based approaches for screening that can be completed by the child or family [29, 36–38]. Screening is recognized as promoting responsive clinical care and providing a roadmap for subsequent interventions tailored to the needs identified [39–42]. Similarly, assessing financial need helps assure that social determinants of health are considered in the delivery of care and allows for the provision of support to promote engagement with the healthcare setting [43–45]. Interventions to address this are available [46–48]. These two Standards are best implemented shortly after diagnosis and near entry into cancer care, although iSTEPPP data suggest that it is equally or more often provided at the point when a problem is identified. Screening for neurocognitive difficulties represents clinical care consistent with established research on neurotoxicity and care that improves educational performance [49, 50]. iSTEPPP data also suggest that neurocognitive screening is not routine and is often provided reactively when a problem is identified.

Monitoring treatment adherence provides needed input as to whether medical treatments are being delivered as intended, essential to overall outcomes and health equity [51]. With the increasing population of childhood cancer survivors, attention to psychosocial needs is one of the key aspects of late effects, including during the transition to adulthood [52]. Other frequently provided services are those related to the patient and the provision of psychoeducation, guidance, and interventions to facilitate coping during treatments and procedures. Some Standards bridge to other services such as palliative care [53] and bereavement programs [54, 55].

The least often provided Standards are those associated with Family and Community. The only statistically significant decline in the frequency of care since 2016 was in three of the four Standards in this group. The lack of psychosocial services for families, in particular parents and siblings, is worrisome and puzzling. Parent behavioral health concerns are prominent in this population [56–58], a caregiver priority [59], and associated with child quality of life [60]. The needs of siblings and the challenges in providing care have been recognized for many years [61, 62]. While understanding that the child is the patient in a pediatric setting, making it more difficult to focus on caregiver concerns, a contextual understanding of children and their development must recognize the importance of supporting parents and other children in the family. Family functioning relates to other Standards, including adherence to treatment [63]. Moreover, parents consistently express an interest in psychosocial care for themselves and siblings [64].

The details with respect to how and when psychosocial care is provided are critically important to understanding the nuances of care delivery. These data are complex and highly variable, but some generalizations can be made. First, the Standards are intended to be universal, proactive, and preventative. Unfortunately, the data suggest that care is often provided inconsistently and in response to an identified problem. The fact that “usually” or “always” was not endorsed more frequently raises questions as to whether all families are receiving comprehensive psychosocial care and whether care is provided with equity to all families [65]. Second, although we recognize that the Standards can be met in many ways, there are evidence-based treatments, supported by recent systematic reviews [66, 67] that are often not being offered [68]. Informal discussion was endorsed frequently across the Standards. While talking with patients and families is necessary in psychosocial care, it is not necessarily sufficient. It should not be used in lieu of evidence-based approaches that may require more specialized staff and additional training or resources to deliver. Moreover, relying on informal and unstandardized approaches may unintentionally communicate that more robust resources are not needed, exacerbating staffing and related challenges.

Alignment with the Standards and perceived quality of care do not differ by the size of programs. The intention across programs likely is to provide excellent and comprehensive care and that providers are working hard to achieve the spirit, if not always the detailed delivery of the Standards. However, there is clearly room for improvement. The frequency and quality of psychosocial care have not changed markedly since the Standards were published, despite a moderate increase in the size of psychosocial teams [24]. These findings are noted in the context of the COVID pandemic and increased mental health needs [69].

With its national scope and inclusion of COG sites from most states where most children receive their care, we intend that the data be used for benchmarking psychosocial care. We were unable to include programs in some rural states. It is also possible that programs with fewer resources or those without integrated psychosocial staff or supportive leadership may not have participated. The COVID-19 pandemic had a wide-ranging global impact on psychosocial care, and on families and providers [70–73] and may have curtailed use of the Matrix and Guidelines. Short- and long-term changes in practice and related influences over this time (e.g., telehealth, eHealth, staff shortages) may have had an ongoing impact on clinical practice, and increased attention to social determinants of health may have also impacted service provision and priorities. We also recognize the limitations of a broad provider survey, including potential biases of providers in rating care, and our inability to “verify” data due to limited information available in the public domain. We would be able to describe psychosocial care more fully had we been able to use a more focused approach to understand details of services provided, including the perspectives of families, and use qualitative methods. We also appreciate that nuances in the questions could be provided. For example, “comprehensive and state-of-the-art” care may be perceived differently and could be confounded by a single-item assessment. Initiatives in Australia, Canada, the Caribbean, and India are underway that will provide a broader perspective on the delivery of care globally and open avenues for cross-nation collaborations.

We conclude with some overarching questions important for future investigation: (i) how can we assure more consistency in psychosocial care within and across programs; (ii) how can all families receive care delivered with preventive rather than reactive approaches; (iii) how can pediatric cancer programs commit to and organize to implement the Standards; (iv) how can we assure a holistic vision of care inclusive of the needs of all members of the family; and (v) what implementation strategies can we introduce to facilitate uptake of the Standards? Recent research is promising in highlighting innovative ways in which psychosocial care can be provided. For example, McTate and colleagues [64] outline a comprehensive caregiver mental health program, Turner and colleagues [3] describe an interdisciplinary, tiered system of supports and services for children with cancer and their families, and Weiler-Wicht et al. [74] have pilot-tested a guide for children throughout their treatment. And providers and centers can access tools [21] to identify approaches and gauge their progress in providing care consistent with the Standards. Much work remains to be done to implement the vision of the Standards, particularly adding the voice of patients and families while also understanding barriers and facilitators of creative and effective approaches.

## Supplementary Material

Supporting Information S1 **Supporting File 1:** pbc31959-sup-0001-SuppMat.docx.

Additional supporting information can be found online in the Supporting Information section.

## Figures and Tables

**TABLE 1:** Mean frequency of care/service provided by psychosocial and medical providers on a 1 (Never) to 5 (Always) scale.

Standard (number)	All (*n* = 168) Mean (SD)	Psychosocial (*n* = 99) Mean (SD)	Medical (*n* = 69) Mean (SD)
*Asking and Monitoring (M = 4.42)*			
Assessment of psychosocial healthcare needs (PSS1)	4.61 (0.61)	4.59 (0.62)	4.65 (0.59)
Assessment of financial need (PSS5)	4.52 (0.67)	4.59 (0.66)	4.41 (0.68)
Monitoring of neurocognitive problems (PSS2)	4.28 (0.91)	4.38 (0.82)	4.15 (1.02)
Adherence to treatment is assessed and monitored (PSS12)	4.41 (0.72)	4.29 (0.80)	4.58 (0.56)
Screening in long-term survivorship (PSS3)	4.24 (0.94)	4.30 (0.91)	4.15 (0.97)
*Intervention and Support (M = 4.25)*			
Psychoeducation, information, anticipatory guidance (PSS7)	4.43 (0.79)	4.52 (0.68)	4.30 (0.91)
Preparatory information about invasive procedures (PSS8)	4.17 (0.81)	4.21 (0.75)	4.12 (0.89)
Psychosocial interventions for invasive procedures (PSS8)	3.81 (0.86)	3.88 (0.85)	3.70 (0.86)
Psychosocial support and Interventions (PSS4)	4.48 (0.67)	4.53 (0.62)	4.39 (0.74)
*Family and Community (M = 3.72)*			
Opportunities for social interaction (PSS9)	3.46 (0.77)	3.37 (0.83)	3.59 (0.66)
Support for school re-entry (PSS11)	4.27 (0.74)	4.29 (0.74)	4.23 (0.75)
Parental mental health (PSS6)	3.84 (0.93)	3.86 (1.01)	3.81 (0.81)
Psychosocial support and interventions for siblings (PSS10)	3.29 (0.84)	3.29 (0.89)	3.28 (0.77)
*Supportive Care and Bereavement (M = 3.88)*			
Palliative care concepts throughout disease process (PSS13)	3.29 (0.87)	3.25 (0.82)	3.34 (0.93)
Developmentally appropriate end-of-life care (PSS13)	4.51 (0.61)	4.47 (0.61)	4.57 (0.61)
Psychosocial care after a child’s death (PSS14)	3.82 (0.96)	3.82 (0.96)	3.82 (0.96)

*Note:* There are no significant differences in mean ratings across medical or psychosocial providers. There are no significant differences by the size of the program.

**TABLE 2:** Approaches used and timing of care related to the psychosocial standards.

Asking and Monitoring				
Standard	Approach used	%	When is care provided	%
Youth with cancer and their family members should routinely receive systematic assessments of their psychosocial healthcare needs (PSS1)	Informal discussion with child/family	85.3	When a problem is identified	79.8
	Structured interview	54.1	First week after diagnosis	68.8
	Psychosocial Assessment Tool (PAT)	25.7	At survivorship visits	61.5
	Other assessment tool or scale	23.9	Diagnosis (within 48 hours)	51.4
	Distress thermometer	18.3	First month after diagnosis	47.7
			Every inpatient admission	46.8
			At end of active treatment	35.8
			Every clinic visit	26.6
			Other specific time points	23.9
Psychosocial staff should assess families’ financial needs, including factors that may influence their access to care (PSS5)	No data collected on approach for this Standard		First week after diagnosis	62.4
			When a problem is identified	60.6
			Diagnosis (within 48 hours)	40.4
			First month after diagnosis	29.4
			Every inpatient admission	25.7
			Other specific time points	18.3
			Every clinic visit	16.5
Patients w/brain tumors and others at high risk for neuropsychological deficits should be monitored for deficits during and after treatment (PSS2)	Referral to neuropsychologist	79.8	When a problem is identified	74.3
	Informal discussion	73.4	At survivorship visits	56.9
	Brief neurocognitive testing	33.0	At end of active treatment	53.2
	Other	11.9	Within 2 months after diagnosis	36.7
			Every clinic visit	28.4
			Every inpatient admission	16.5
			Other specific time points	15.6
Adherence should be assessed routinely and monitored throughout treatment (PSS12)^a^	Ask family members verbally	84.4		
	Ask patient verbally	82.6		
	Self-report (by the patient)	67.0		
	Self-report (by the parents)	67.0		
	Provider report (staff)	63.3		
	Ask medical providers verbally	58.7		
	Blood test	56.9		
	Medication log	39.4		
	Electronic monitoring device	8.3		
	Other	6.4		
Psychosocial staff should monitor long-term childhood cancer survivors (PSS3)^a^	Informal discussion w/child/family	73.4		
	Screen for distress, anxiety, depression	57.8		
	Screen educational/vocational progress	55.0		
	Screen for social/relationship difficulties	52.3		
	Other	8.3		
Interventions and Support				
Standard	Approach used	%	When is care provided	%
Psychosocial staff should provide psychoeducation, information, and anticipatory guidance regarding the child’s disease and treatment (PSS7)^a^	Meet with child and family	86.2		
	Meet with parent(s) wo child	81.7		
	Provide written materials	80.7		
	Meet individually with child	79.8		
	Provide access to resources online	73.4		
	Provide informational videos	33.0		
	Other	8.3		
Psychosocial staff should provide preparatory information related to invasive medical procedures (PSS8a)^a^	Meet with child and family	81.7		
	Meet individually with child	77.1		
	Meet with parent(s) without child	67.0		
	Provide written materials	61.5		
	Provide informational videos	44.0		
	Provide access to resources online	41.3		
	Other	10.1		
Psychosocial staff should provide psychological interventions for invasive medical procedures (PSS8b)^a^	Distraction	84.4		
	Relaxation	79.8		
	Cognitive/Behavioral therapy	66.1		
	Other	11.9		
	Hypnosis	9.2		
Psychosocial staff should provide youth with cancer and their families with psychosocial support and intervention (PSS4)	Informal discussion child & family	85.3	When a problem is identified	78.9
	Supportive psychotherapy	79.8	Within the first week	66.1
	Cognitive/Behavioral therapy	69.7	Within the first month	56.9
	Pharmacologic treatment	61.5	At diagnosis (within 48 hours)	55.0
	Problem solving skills training (PSST)	35.8	At survivorship visits	49.5
	Family Therapy	33.9	At every inpatient admission	41.3
	Support groups	28.4	At the end of active treatment	40.4
	Other	15.6	At every clinic visit	25.7
	SCCIP	4.6	At other specific time points	12.8
Family and Community				
Standard	Approach used	%	When is care provided	%
Psychosocial staff should provide youth with cancer with opportunities for social interaction (PSS9)^a^	Camps	68.8		
	Facilitated activities/programs	62.4		
	Support groups	22.9		
	Other	22.9		
	Online groups/chat rooms	20.2		
Psychosocial staff should provide patients with support for re-entry into school (PSS11)^a^	Staff person who coordinates w school	68.8		
	In-house school program	45.9		
	Other school services	19.3		
			
Psychosocial staff should assess the mental health needs of parents/caregivers (PSS6)	Informal discussion w parents	80.7	When a problem is identified	71.6
	Referral to therapist in community	76.1	Within the first week	56.9
	Referral to community psychiatrist	60.6	Within the first month	48.6
	Supportive psychotherapy	56.9	At diagnosis (within 48 hours)	42.2
	Referral to therapist in hospital	39.4	At survivorship visits	31.2
	Referral to psychiatrist in hospital	33.9	At every inpatient admission	30.3
	Cognitive/Behavioral Therapy	31.2	At end of active treatment	25.7
	Family therapy	25.7	At every clinic visit	17.4
	Support groups	23.9	At other specific time points	8.3
	Problem solving skills training	19.3		
	Couples/Marital Counseling	11.9		
	Other	6.4		
	SCCIP	1.8		
Psychosocial staff should provide psychosocial support and intervention for siblings of patients (PSS10)^a^	Informal discussion w sibs/family	78.0		
	Referral community providers	66.1		
	Resource materials to parents	59.6		
	Supportive psychotherapy siblings	35.8		
	Sibling programs	33.0		
	Sibling support groups	12.8		
	Other	7.3		
Supportive Care and Bereavement				
Standard	Approach used	%	When is care provided	%
Psychosocial staff should deliver care after a child’s death (PSS14)	Phone call to family to assess needs	72.5		
	Phone call to family to resources	70.6		
	Letter or card	67.9		
	Phone call to assess psychosocial status	57.8		
	Legacy items	56.0		
	Memorial programs at hospital	54.1		
	Support groups	21.1		
	In person meeting with family	20.2		
	Psychotherapy	18.3		
	Other	9.2		

a Questions about timing were asked for a subset of Standards.

**TABLE 3:** Mean frequency of psychosocial care provided by Standard in 2016 (PIPS-CSS) and 2023 (iSTEPPP) studies on a 1 (Never) to 5 (Always) scale.

Standard (number)	PIPS-CSS (*n* = 231) Mean (SD)	iSTEPPP^a^ (*n* = 168) Mean (SD)
*Asking and Monitoring*		
Assessment of psychosocial healthcare needs (PSS1)	4.62 (0.67)	4.61 (0.61)
Assessment of financial need (at diagnosis) (PSS5)	4.47 (0.83)	4.52 (0.67)
Monitoring of neurocognitive problems (PSS2)	4.15 (0.99)	4.28 (0.91)
Adherence to treatment is assessed and monitored (PSS12)	4.44 (0.74)	4.41 (0.72)
Screening in long-term survivorship (PSS3)	4.01 (1.06)	4.24 (0.94)
*Intervention and Support*		
Psychoeducation, information, anticipatory guidance (PSS7)	4.36 (0.77)	4.43 (0.79)
Preparatory information about invasive procedures (PSS8)	4.14 (0.90)	4.17 (0.81)
Psychosocial interventions for invasive procedures (PSS8)	3.88 (0.91)	3.81 (0.86)
Psychosocial support and interventions (PSS4)	4.53 (0.66)	4.48 (0.67)
*Family and Community*		
Opportunities for social interaction (PSS9)^b^	3.96 (0.82)	3.46 (0.77)
Support for school re-entry (PSS11)	4.29 (0.78)	4.27 (0.74)
Parental mental health (PSS6)^b^	4.23 (0.76)	3.84 (0.93)
Psychosocial support and interventions for siblings (PSS10)^b^	3.65 (0.87)	3.29 (0.84)
*Supportive Care and Bereavement*		
Palliative care concepts throughout disease process (PSS13a)	3.19 (0.98)	3.29 (0.87)
Developmentally appropriate end-of-life care (PSS13b)	4.56 (0.65)	4.51 (0.61)
Psychosocial care after a child’s death (PSS14)	3.86 (0.94)	3.82 (0.96)

*Note: p* < 0.001.

a There are no significant differences by size of program.

b Significant difference in mean score between PIPS-CSS (2016) and iSTEPPP (2023).

## Abbreviations:

COG: Children’s Oncology Group
iSTEPPP: Implementing the Standards Together—Engaging Parents and Providers in Psychosocial Care
NCCN: National Comprehensive Cancer Network
PIPS-CSS: Preparing to Implement Psychosocial Standards: Current Services and Staffing
PSS: Psychosocial Standard
PSS1: Standard 1 Assessment of psychosocial healthcare needs
PSS2: Standard 2 Monitoring of neuropsychological deficits
PSS3: Standard 3 Screening in long-term survivorship
PSS4: Standard 4 Psychosocial support and interventions
PSS5: Standard 5 Assessment of financial need
PSS6: Standard 6 Parental mental health
PSS7: Standard 7 Psychoeducation, information, and anticipatory guidance
PSS8: Standard 8 Preparatory information for procedures
PSS9: Standard 9 Opportunities for social interaction
PSS10: Standard 10 Siblings
PSS11: Standard 11 School support
PSS12: Standard 12 Adherence
PSS13: Standard 13 Palliative care/end-of-life care
PSS14: Standard 14 Bereavement care
REDCap: Research Electronic Data Capture
Standards: Standards for Psychosocial Care for Children with Cancer and Their Families
US: United States
