# Supplementary material for: Delivering Care Consistent With the Psychosocial Standards—Provider Report: Implementing the Standards Together—Engaging Parents and Providers in Psychosocial Care (iSTEPPP) Study
Source: Pediatr Blood Cancer. Author manuscript; Available in PMC 2026 Jul 24. (PMC13399412; doi:10.1002/pbc.31959)
Supplement: Supporting Information S1 — Supporting File 1: pbc31959-sup-0001-SuppMat.docx. [file NIHMS2186128-supplement-Supporting_Information_S1.docx]

**Standards for the Psychosocial Care of Children With Cancer and Their Families^[[1]](#footnote-1)^**

Fifteen evidence based standards for the psychosocial care of children with cancer were published in *Pediatric Blood and Cancer* in December 2015. <http://onlinelibrary.wiley.com/doi/10.1002/pbc.v62.S5/issuetoc>.

1. Youth with cancer and their family members should routinely receive systematic assessments of their psychosocial health care needs.
2. Patients with brain tumors and others at high risk for neuropsychological deficits as a result of cancer treatment should be monitored for neuropsychological deficits during and after treatment.
3. Long-term survivors of child and adolescent cancers should receive yearly psychosocial screening for: a) adverse educational and/or vocational progress, social and relationship difficulties; b) distress, anxiety, and depression and c) risky health behaviors. Adolescent and young adult survivors and their parents should receive anticipatory guidance on the need for life long follow-up care by the time treatment ends and repeated at each follow-up visit.
4. Youth with cancer and their family members should have access to psychosocial support and interventions throughout the cancer trajectory and access to psychiatry as needed.
5. Pediatric oncology families are at high risk for financial burden during cancer treatment with associated negative implications for quality of life and parental emotional health. Assessment of risk for financial hardship should be incorporated at time of diagnosis for all pediatric oncology families. Domains of assessment should include risk factors for financial hardship during therapy including: pre-existing low-income or financial hardship, single parent status, distance from treating center, anticipated long/intense treatment protocol, and parental employment status. Targeted referral for financial counseling and supportive resources (including both governmental and charitable supports) should be offered based on results of family assessment. Longitudinal reassessment and intervention should occur throughout the cancer treatment trajectory and into survivorship or bereavement.
6. Parents and caregivers of children with cancer should have early and ongoing assessment of their mental health needs. Access to appropriate interventions for parents and caregivers should be facilitated to optimize parent, child and family well-being.
7. Youth with cancer and their family members should be provided with psychoeducation, information, and anticipatory guidance related to disease, treatment, acute and long-term effects, hospitalization, procedures, and psychosocial adaptation. Guidance should be tailored to the specific needs and preferences of individual patients and families and be provided throughout the trajectory of cancer care.
8. Youth with cancer should receive developmentally appropriate preparatory information about invasive medical procedures. All youth should receive psychological intervention for invasive medical procedures.
9. Children and adolescents with cancer should be provided opportunities for social interaction during cancer therapy and into survivorship following careful consideration of the patients’ unique characteristics, including developmental level, preferences for social interaction, and health status. The patient, parent(s) and a psychosocial team member (e.g., designee from child life, psychology, social work, or nursing) should participate in this evaluation at time of diagnosis, throughout treatment and when the patient enters survivorship; it may be helpful to include school personnel or additional providers.
10. Siblings of children with cancer are a psychosocially at-risk group and should be provided with appropriate supportive services. Parents and professionals should be advised about ways to anticipate and meet siblings’ needs, especially when siblings are unable to visit the hospital regularly.
11. In collaboration with parents, school-age youth diagnosed with cancer should receive school reentry support that focuses on providing information to school personnel about the patient’s diagnosis, treatment, and implications for the school environment and provides recommendations to support the child’s school experience. Pediatric oncology programs should identify a team member with the requisite knowledge and skills who will coordinate communication between the patient/family, school, and the health care team.
12. Adherence should be assessed routinely and monitored throughout treatment.
13. Youth with cancer and their families should be introduced to palliative care concepts to reduce suffering throughout the disease process regardless of disease status. When necessary youth and families should receive developmentally appropriate end of life care, which includes bereavement care after the child’s death. A member of the health care team should contact the family after a child’s death to assess family needs, to identify those for negative psychosocial sequelae, to continue care, and to provide resources for bereavement support.
14. A member of the healthcare team should contact the family after a child’s death to assess family needs, to identify those at risk for negative psychosocial sequelae, to continue care, and to provide resources for bereavement support.
15. Open, respectful communication and collaboration among medical and psychosocial providers, patients and families is essential to effective patient- and family-centered care. Psychosocial professionals should be integrated into pediatric oncology care settings as integral team members and be participants in-patient care rounds/meetings. Pediatric psychosocial providers should have access to medical records and relevant reports should be shared among care team professionals, with psychological report interpretation provided by psychosocial providers to staff and patients/families for patient care planning. Psychosocial providers should follow documentation policies of the health system where they practice in accordance with ethical requirements of their profession and state/federal laws. Pediatric psychosocial providers must have specialized training and education and be credentialed in their discipline to provide developmentally-appropriate assessment and treatment for children with cancer and their families. Experience working with children with serious, chronic illness is crucial as well as ongoing relevant supervision/peer support.


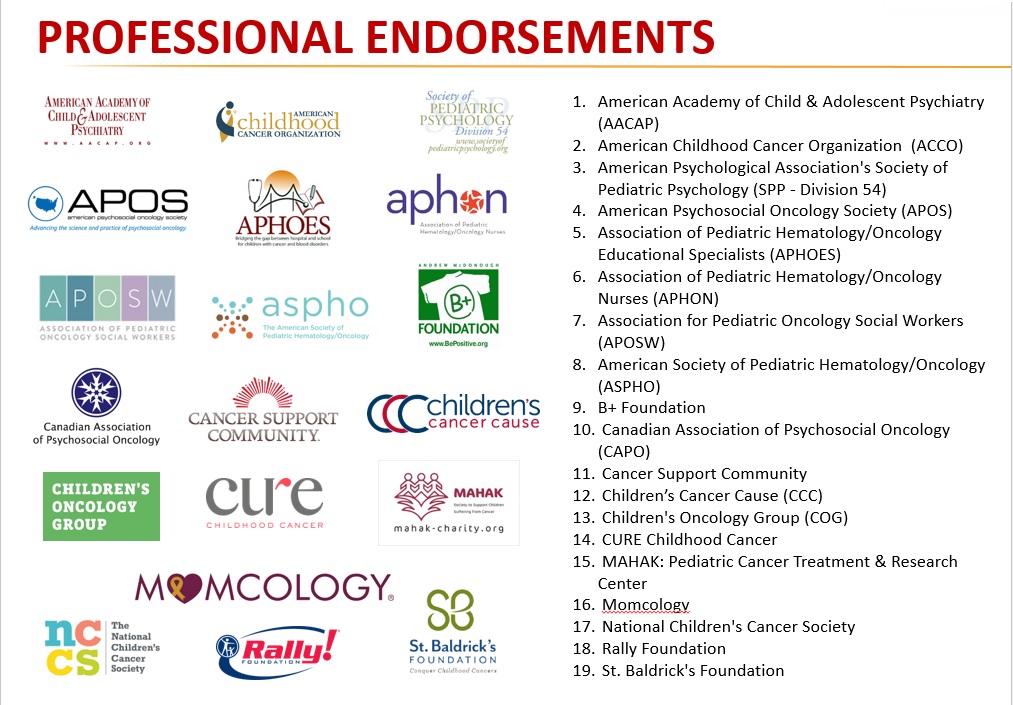


1. From Wiener L, Kazak AE, Noll RB, Patenaude AF, Kupst MJ. Standards for the Psychosocial Care of Children With Cancer and Their Families: An Introduction to the Special Issue. *Pediatr Blood Cancer*. 2015 Dec;62 Suppl 5:S419-24. doi: 10.1002/pbc.25675. Each standard is published in a systematic review in the special issue. [↑](#footnote-ref-1)
